# Effect of Intraoperative High-Dose Remifentanil on Postoperative Pain: A Prospective, Double Blind, Randomized Clinical Trial

**DOI:** 10.1371/journal.pone.0091454

**Published:** 2014-03-25

**Authors:** Yan-Ling Zhang, Peng Ou, Xiang-Hang Lu, Yan-Ping Chen, Jun-Mei Xu, Ru-Ping Dai

**Affiliations:** Department of Anesthesiology, The Second Xiangya Hospital of Central South University, Changsha, Hunan Province, China; San Raffaele Scientific Institute, Italy

## Abstract

**Background:**

Remifentanil, an ultra-short-acting opioid, is widely used for pain control during surgery. However, regular dose (RD) remifentanil exacerbates postoperative pain in a dose-dependent manner. Recent studies suggest that high-dose (HD) remifentanil offers sustained analgesia in experimental studies. We thus hypothesized that intraoperative administration of high-dose remifentanil may attenuate postoperative pain.

**Methods:**

In this prospective, randomized, double blind, controlled clinical study, sixty patients undergoing thyroidectomy (18–60 years-of-age) received an intraoperative infusion of 0.2 (RD group) or 1.2 μg kg^−1^min^−1^ (HD group) remifentanil during thyroidectomy. A visual analogue scale (VAS) was used to measure pain intensity. Mechanical pain threshold on the forearm was assessed using von Frey filaments before surgery (baseline), 2 h postoperatively and 18–24 h postoperatively. The primary outcome was to compare the difference of VAS score at different time points after operation and morphine consumption 24 h postoperatively between RD and HD groups. The second outcome was to compare the difference of mechanical pain thresholds in the forearm postoperatively between RD and the HD groups.

**Results:**

VAS scores were lower 30 min postoperatively in the HD group (1.29±1.67, 95% CI 0.64–1.94) compared with the RD group (2.21±1.67, 95% CI 1.57–2.84) (t = 3.427, p = 0.0043, RD group vs. HD group). Postoperative morphine consumption was much lower in the HD group compared with the RD group (1.27±1.88 mg vs. 0.35±1.25 mg, p = 0.033). In both groups, mechanical pain threshold was decreased 18–24 h postoperatively (2.93±0.209 Ln(g) vs. 3.454±2.072 Ln(g), p = 0.032 in RD group; 2.910±0.196 Ln(g) vs. 3.621±0.198 Ln(g), p = 0.006 in HD group, 18–24 h postoperatively vs baseline).

**Conclusions:**

Intraoperative administration of high-dose remifentanil decreased VAS scores and morphine consumption postoperatively. Thus, modulation of intraoperative opiates may be a simple and effective method of postoperative pain management.

**Trial Registration:**

This trial is registered in ClinicalTrials.gov, with the Name: Effect of Higher Doses of Remifentanil on Postoperative Pain in Patients Undergoing Thyroidectomy, and ID number: NCT01761149.

## Introduction

Remifentanil, a potent ultra-short-acting μ-opioid agonist, is a widely used analgesic in clinical anesthesia due to its rapid onset of action and clearance after withdrawal. However, remifentanil, like other μ-opioid receptor agonists, exacerbates postoperative pain as indicated by enhanced pain sensitivity, increased postoperative pain scores, and opioid consumption during remifentanil withdrawal [1], [2]. Within a clinically regular dose range, remifentanil-induced hyperalgesia is dose-dependent. For example, in a previous study, relatively high-dose (HD) remifentanil (0.4 μg kg^−1^min^−1^) increased postoperative pain and morphine consumption, whereas low-dose remifentanil (0.05 μg kg^−1^min^−1^) had no effect on postoperative pain sensation or opioid consumption [3]. In a more recent study, an intraoperative infusion of remifentanil (0.2 μg kg^−1^min^−1^) caused greater pain sensitivity and greater visual analogue scale (VAS) scores than in those who received an infusion of 0.05 μg kg^−1^min^−1^ [4]. In another recent study, investigators reported that after laparoscopic ureteroneocystostomy, pediatric patients who intraoperatively received 0.6 μg kg^−1^min^−1^ and 0.9 μg kg^−1^min^−1^ of remifentanil required more fentanyl postoperatively than those who received saline or 0.3 μg kg^−1^min^−1^ remifentanil [5]. Thus, doses of remifentanil beyond clinically regular doses may exacerbate postoperative pain compared to lower doses.

Interestingly, an experimental study by Drdla-Schutting and colleagues revealed that brief application of HD remifentanil reversed various forms of activity-dependent long-term potentiation (LTP) at C-fiber synapses, a synaptic model of hyperalgesia [6]. In their study, conditioning low-frequency stimulation (LFS) of sciatic nerve C-fibers induced spinal LTP of C-fiber evoked field potentials in adult rats. A brief intravenous infusion of HD remifentanil (450 μg kg^−1^h^−1^) prevented the LFS-evoked spinal LTP of C-fiber evoked field potential. However, relatively low-dose remifentanil (225 μg kg^−1^h^−1^) infusion could not reverse the activated spinal LTP of C-fiber evoked field potentials. Furthermore, a 1-h infusion of HD remifentanil inhibited capsaicin-induced mechanical allodynia for more than 6 h [6]. These findings suggest that, in contrast to withdrawal-evoked hyperalgesia in response to RD remifentanil, higher doses may persistently inhibit injury-induced hyperalgesia after withdrawal. Consistent with these findings, another study confirmed that infusion of 20 μg kg^−1^min^−1^ remifentanil for 20 min inhibited thermal hyperalgesia in a neuropathic pain model [7]. However, whether infusion of higher doses of remifentanil exerts a similar persistently analgesic effect on surgical pain in clinical practice is uncertain. Given that remifentanil is widely used to provide analgesia during surgery, modulation of intraoperative remifentanil may be a novel approach to relieving postoperative pain.

The present study hypothesized that intraoperative infusion of HD remifentanil attenuated postoperative pain and aimed to test this hypothesis in the patients undergoing thyroidectomy. Here we showed that HD remifentanil decreased VAS scores and reduced morphine consumption postoperatively. However, HD remifentanil still reduced mechanical pain thresholds in the forearm, a site remote from the surgical site. Thus, the present study suggested that different mechanisms may regulate the effect of remifentanil on pain sensation in intact sites relative to injured sites; and intraoperative modulation of high dose opioids may offer a convenient and effective technique for postoperative pain control.

## Materials and Methods

### Subjects

The protocol for this trial and supporting CONSORT checklist are available as supporting information; see Checklist S1 and Protocol S1. This clinical trial was approved by the ethics committee of Second Xiangya Hospital of Central South University and registered with Clinicaltrial.gov, with ID number: NCT01761149. We obtained written informed consent from all enrolled participants. Sixty patients (18–60 years-of-age) with an ASA status of I or II undergoing thyroidectomy were enrolled between December 2012 and March 2013. All patients underwent open thyroidectomy under general anesthesia. Exclusion criteria included a history of chronic pain, drug abuse or chronic use of opioids or sedative drugs, psychiatric or neurologic disease, obesity (body mass index, BMI>30), a history of neck surgery, intake of any analgesic drug within 48 h prior to surgery, and re-operation.

### Experimental Procedure and Design

On the day before surgery, baseline mechanical pain thresholds were measured in a delimited area of 3×3 cm^2^ in the left central volar forearm and the visual analogue scale (VAS:0 = no pain,10 = the worst pain imaginable) was shown to the patients. A set of 10 hand-held Von Frey monofilaments calibrated to deliver an increasing force on the skin from 4 to 300 g was used for this purpose (Touch-test Sensory Evaluator, North Coast Medical Inc, CA, USA) as described previously [3], [5]. When patients were relaxed with eyes closed, in quiet surroundings, the filaments were placed perpendicularly against the skin surface until the filaments bent, and were held in place for 1–1.5 s. An interval of 15 s was allowed between trials. The mechanical pain threshold was defined as the smallest force that was interpreted as painful by the patient. All patients received atropine 0.5 mg and phenobarbital 0.1 g (IM) 30 min before surgery. In the operating theatre, standard monitoring (ECG, pulse oximeter, non-invasive arterial blood-pressure measured in the right arm) and a bispectral index (BIS; Bispectral indexTM, Aspect Medical System, Norwood, MA, USA) were performed and the baseline values were recorded. Ringer’s solution was continuously infused intravenously to maintain blood volume.

Anesthesia was induced with continuous propofol infusion and remifentanil by target-controlled infusion (1 ng ml^−1^) based on the BIS value. Once the BIS value was below 60, 0.15 mg kg^−1^ cisatracurium was administered to facilitate tracheal intubation. After tracheal intubation, patients were ventilated with 40% oxygen without any inhaled anesthetics. Maintenance of anesthesia was achieved with propofol only by target controlled infusion to maintain BIS values between 40 and 60.

Patients were randomly assigned, in a double-blind manner, to one of two groups (30 patients per group). Before the study began, a random-number table was constructed. For each patient, an envelope containing the group assignment was prepared, sealed, and sequentially numbered. On the day of surgery and before induction of anesthesia, an anesthesiologist not involved in the evaluation of the patient opened the patient’s envelope and prepared remifentanil syringes. The patients and the anesthesiologists involved in assessing postoperative pain, morphine consumption, VAS data collection and data analyses were unaware of any group assignment. In case of emergency, the attending anesthesiologist was allowed to break the code.

The two treatment groups were as follows:

Clinical-regular-dose remifentanil (RD): patients were administered with remifentanil at a rate of 0.2 μg kg^−1^min^−1^ starting from 1 minute prior to skin incision to the completion of surgery.

High-dose remifentanil (HD): The procedure was similar to the HD group except that the dose was 1.2 μg kg^−1^min^−1^.

In the case of hypotension, defined by a systolic arterial pressure less than 80 mm Hg or a mean arterial pressure less than 60 mm Hg, ephedrine (5 mg, iv) was given and additional intravenous fluids were administered as needed by the anesthesiologist. If the patient’s heart rate was less than 50 beats per min, then atropine (0.3 mg) was administered to maintain a heart rate greater than 50 beats per min.

All patients were transferred to the post-anesthesia care unit and extubated there. An anesthesiologist, blinded to the patients’ group assignment, evaluated the VAS score in the post-anesthesia care unit at 30, 45, 60, 75, 90, 120 min and 18–24 h postoperatively. When the patients described VAS ≥4, morphine was infused (0.05 mg/kg, iv, at 15-min intervals) until the VAS<4. Patient-controlled analgesia was not used because of its rarity in this clinical situation. Postoperative nausea and vomiting were prevented with ondansetron (10 mg, iv) after surgery was concluded.

Postoperative mechanical pain thresholds were measured at 2 h and 18–24 h after surgery in a manner identical to that described previously. To avoid inter-rater variations, all pain measurements were performed by one trained investigator.

The primary outcome was to compare the difference of VAS score at 30 min, 45 min, 60 min, 90 min, 2 h and 18–24 h after operation and cumulative morphine consumption 24 h postoperatively between the RD group and the HD group. The second outcome was to compare the difference of mechanical pain thresholds in the forearm at 2 h and 18–24 h postoperatively between the RD group and the HD group.

### Statistical Analysis

In the preliminary study (unpublished data), VAS scores were 2.32±0.33 for the RD group and 1.44±0.45 for the HD group 30 min postoperatively. The estimated sample size was 4 patients per group with a β-risk of 80% at an α-level of 0.05 for detecting a difference in postoperative VAS at least of 0.8 at 30 min postoperatively. In contrast, baseline mechanical pain thresholds for the forearm were 3.6±0.8 Ln(g), and 3.0±0.8 Ln (g) at 24 h after surgery in the HD group (values are Ln of force in mg) [8]. The estimated sample size was 28 patients per group with a β-risk of 80% at an α-level of 0.05 for detecting a difference in mechanical sensory threshold at least of 0.8 at 24 h after operation when compared to baseline. Thus, 30 patients were enrolled in each group to investigate the effect of HD remifentanil on mechanical pain sensation, and this sample size permitted sufficient power to detect statistically significant differences even with some patients drop outs.

Age, weight, height, BMI, duration of surgery, intraoperative propofol consumption and postoperative morphine consumption were analyzed with an unpaired *t* test. Hemodynamic parameters including systolic (SBP), diastolic (DBP) and mean arterial pressure (MAP), heart rate (HR), the BIS scale, mechanical pain thresholds and VAS scores were analyzed by two-way ANOVA for inter-group comparisons. In cases of statistical significance, *post hoc* tests were conducted with a Bonferroni’s adjustment. The χ^2^ test was used to compare gender, ASA levels, number of patients requiring ephedrine or morphine, and postoperative complications (postoperative nausea and vomiting, respiratory depression, shivering). All analyses were performed using SPSS 13.0 and GraphPad Prism 5.

## Results

**Figure 1:**
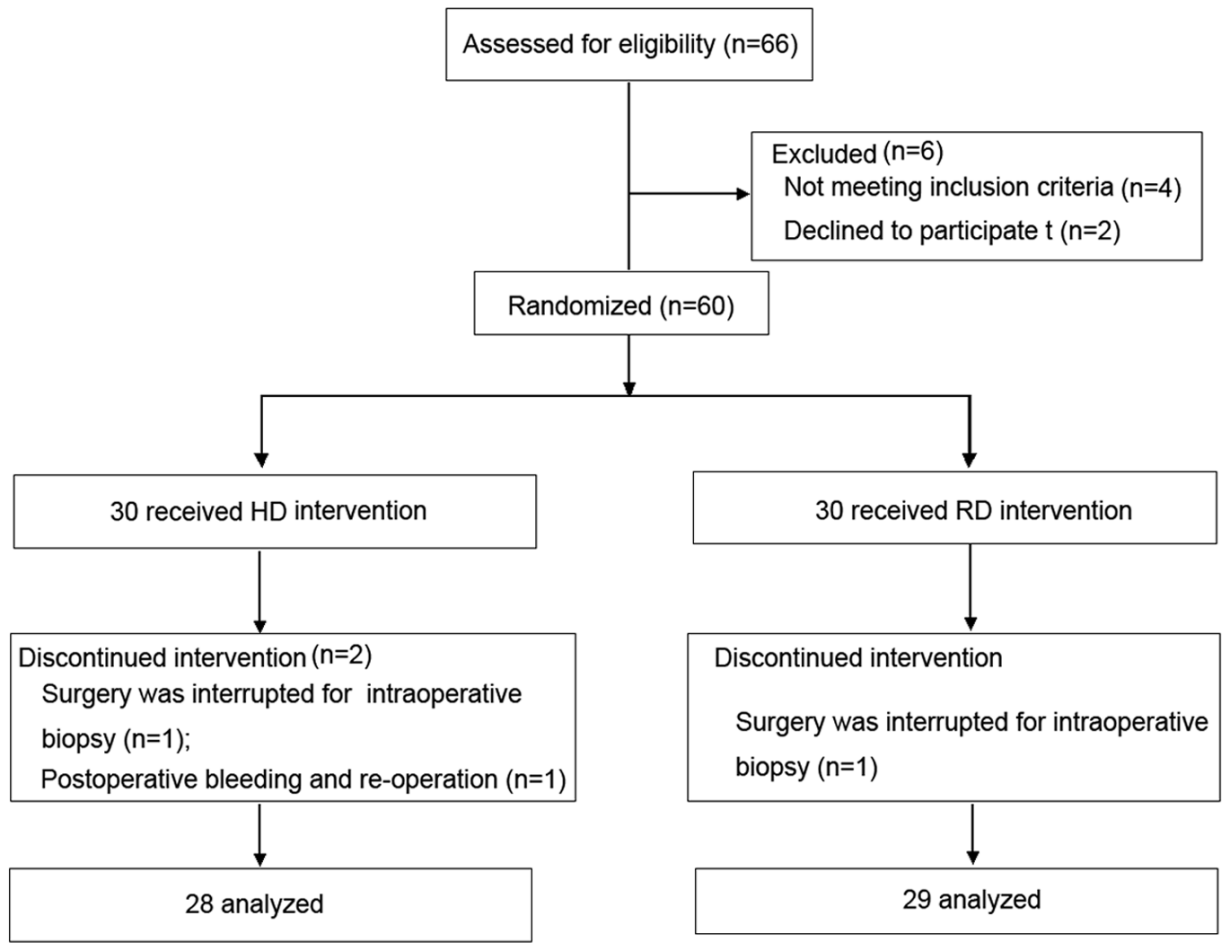
Trial profile.

Figure 1 depicts the trial enrollment (N = 57). Of the three patients excluded, one had postoperative bleeding and required re-operation; two had an interrupted surgery for an intraoperative biopsy, which lasted more than 1 h. Thus, of the 57 enrolled, 29 patients were randomized to the RD group; 28 patients were randomized to the HD group. Table 1 depicts patient baseline characteristics, and these were comparable for both treatment groups.

**Table 1:** Patients’ characteristics.

	RD (29)	HD (28)
Age (yr)	41.6±12.2	37.3±9.8
Gender (M/F)	9/20	8/20
ASA (I/II)	18/11	18/10
Height (m)	1.60±0.07	1.63±0.07
Weight (kg)	58.0±12.7	60.5±12.3
BMI	22.5±3.50	22.6±3.26
Duration of surgery (min)	92.1±28.5	85.0±44.3

Values are presented as mean±SD RD, regular-dose group; HD, high-dose group.

**Figure 2:**
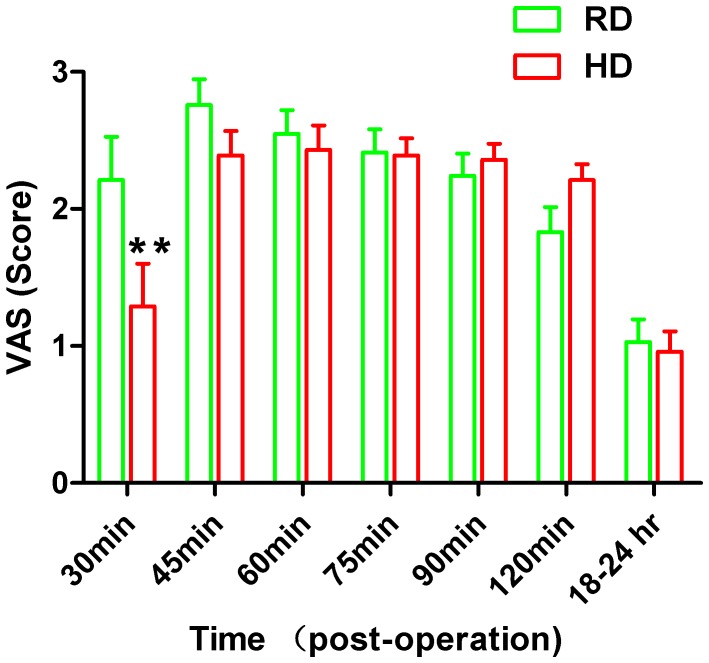
VAS scored at different times postoperatively. Note the VAS score at 30(p<0.01, Two-way ANOVA analysis followed by Bonferroni’s *post hoc* test). **, p<0.01, RD group *vs* HD group.

As shown in Figure 2, there were significant difference in the VAS score at the different time points postoperatively between the RD and the HD groups (F_(6,385)_ = 2.356, p = 0.0236). At 30 min postoperatively, VAS scores were significantly higher in the RD group (2.21±1.67, 95% CI 1.57–2.84) compared to the HD group (1.29±1.67, 95% CI 0.64–1.94) (t = 3.427, p = 0.0043, Two-way ANOVA followed by Bonferroni’s test). At later time points, no significant differences were observed between VAS scores in these two groups.

In the RD group, ten of 29 patients required morphine treatment, but only two of 28 patients in the HD group required morphine treatment (p = 0.027; Table 2). Morphine consumption 24 h postoperatively was greater in the RD group compared to the HD group (1.27±1.88 mg vs. 0.35±1.25 mg, p = 0.033).

**Table 2:** Patients’ anesthetic characteristics.

	RD(29)	HD(28)	P-value
Waking time (min)	13.3±2.7	12.6±3.3	0.356
Extubation time (min)	15.2±2.9	14.1±1.7	0.326
Propofol consumption (mg)	702.5±213.3	636.3±221.5	0.253
Morphine consumption (mg)	1.27±1.88	0.35±1.25	0.033
Morphine (No. of patients)	10	2	0.027
Ephedrine (No. of patients)	2	5	0.180
PONV	1	2	0.245

Values are presented as mean±SD, or the number of patients. PONV, postoperative nausea or vomiting.

**Figure 3:**
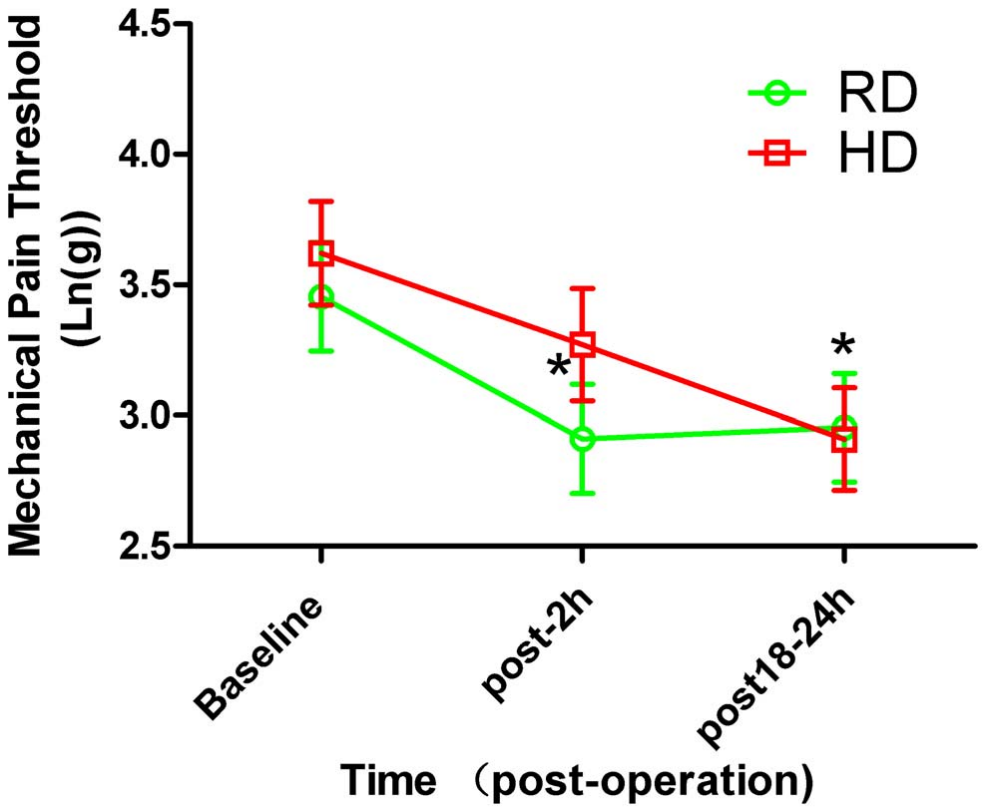
Time course of mechanical pain threshold in the forearm after surgery. Note the significantly decreased mechanical pain threshold at 2–24 h postoperatively compared to baseline in RD group (p<0.05, Two-way ANOVA analysis followed by Bonferroni’s *post hoc* test). In HD group, the mechanical pain threshold is also significantly decreased at 18–24 h postoperatively when compared with baseline (p<0.01, Two-way ANOVA analysis followed by Bonferroni’s *post hoc* test). *p<0.05, post-2 h or 18–24 h postoperatively *vs* baseline.

Figure 3 depicts baseline mechanical pain thresholds for the forearm, 2 h and 18–24 h postoperatively. Two-way ANOVA analysis showed that there was significant difference in the mechanical threshold at different time point postoperatively in both groups (F_(2,104)_ = 15.40, p<0.0001). Compared with baseline, mechanical pain thresholds were significantly lower at 18–24 h postoperatively in both groups (2.93±0.209 Ln (g) vs. 3.454±0.207 Ln (g), p = 0.032 in the RD group; 2.910±0.196 Ln (g) vs. 3.621±0.198 Ln (g), p = 0.006 in the HD group, 18–24 h postoperatively vs baseline, two-way ANOVA followed by Bonferroni’s test). In the HD group, mechanical pain thresholds at baseline and 2 h postoperatively did not differ significantly (t = 1.208, p>0.05). However, in the RD group, mechanical pain thresholds were significantly lower 2 h postoperatively (2.910±0.203 Ln (g)) compared with baseline (3.454±0.2072 Ln (g)) (t = 3.201, p = 0.0322, post-2 h vs baseline). *Post hoc* Bonferroni’s test also showed that no significant difference in the mechanical pain threshold in the post-operative 2 h (t = 1.297, p>0.05) and 18–24 h (t = 0.2392, p>0.05) between the RD and the HD groups.

No significant difference was observed in propofol consumption, waking time or extubation time in these two groups. Patients requiring ephedrine treatment did not differ significantly between the RD and the HD group. Two patients in the HD group and one patient in the RD group reported postoperative nausea or vomiting (Table 2).

Table 3 shows hemodynamic variables during surgery in the two treatment groups. Intraoperatively, SBP, DBP and MAP, and HR were similar across the indicated time points for both groups. BIS values were also comparable for both groups.

**Table 3:** Hemodynamic data and BIS scales.

	HR		SBP(mmHg)		DBP(mmHg)		MAP(mmHg)		SpO_2_(%)		BIS	
	RD	HD	RD	HD	RD	HD	RD	HD	RD	HD	RD	HD
**Basal**	79±13	75±14	124±17	119±16	75±13	75±8	89±14	86±10	99±1	99±1	95±2	95±4
**Induction**	77±13	73±14	105±16	105±13	65±11	65±12	76±13	76±12	99.6±0.7	99.8±0.5	49±6	50±11
**Aft indu**	77±10	73±15	108±20	112±15	69±13	71±12	82±17	83±13	99.7±0.5	99.9±0.3	54±12	50±9
**5 min aft**	77±11	73±14	107±19	109±15	69±13	66±12	80±17	78±12	99.8±0.6	99.7±0.8	59±10	52±6
**10 min aft**	74±9	70±13	108±23	110±18	71±15	68±13	82±18	80±15	99.9±0.3	99.9±0.2	57±10	52±3
**End oper**	69±11	62±10	111±17	102±18	73±12	62±13	84±15	73±15	100±0	100±0	46±5	40±10
**5 min extu**	78±12	81±14	127±18	119±15	78±13	76±7	96±16	89±13	100±0	99.2±1.4		

Values are expressed as mean±SD. RD: regular dose remifentanil group; HD: high-dose remifentanil group; HR: heart rate; SBP: systolic blood pressure; DBP: diastolic blood pressure; MAP: mean arterial pressure; BIS: bispectral index; Aft indu: 1 min after induction; 5 min aft and 10 min aft: 5 min and 10 min after induction, respectively; End oper: End of operation; and 5 min extu: 5 min after extubation. BIS values at 5 min after extubation were often missing, so no comparative analysis was conducted.

## Discussion

The present study has made two important findings. First, HD remifentanil attenuated early postoperative VAS scores and reduced morphine consumption after mildly painful surgery (thyroidectomy), indicating that intraoperative HD remifentanil exerts a persistent analgesic effect, which may be a novel strategy for postoperative pain control. Second, HD remifentanil also reduced mechanical pain thresholds of the forearm, a site remote from the surgical site, implying that different mechanisms regulate the effect of remifentanil on pain sensation in intact sites relative to injured sites.

This HD dosage was based on a recent experimental study in which 450 μg kg^−1^h^−1^ remifentanil was continuously infused in rats to block capsaicin-evoked hyperalgesia [6]. Normally, the dose used in rats is ∼6.1 times that used in humans (72 μg kg^−1^h^−1^ as used in this study) [9]. Here, intraoperative infusion of HD remifentanil decreased VAS scores at 30 min postoperatively and reduced morphine consumption after surgery compared to RD remifentanil. The sustained analgesic effect of HD remifentanil is unlikely due to the residual plasma concentrations of remifentanil after withdrawal. Remifentanil has a very short elimination half-life (3–6 min) which is not increased with prolonged infusion [10]. Although we did not measure the effect of lower dose (0.05 μg kg^−1^min^−1^) remifentanil on postoperative pain, previous studies indicated that patients receiving an intraoperative infusion of 0.05 μg kg^−1^min^−1^ remifentanil during thyroidectomy had high VAS scores 6 h postoperatively and that 70% of those patients required analgesics [4]. Together with previous studies indicating that remifentanil-induced hyperalgesia is dose-dependent [3]–[5], our findings further suggested that the dose-dependent manner of remifentanil-evoked hyperalgesia is within a range of dosage. When the dose administered exceeded this range, remifentanil would not increase, but decrease postoperative VAS score and reduce postoperative morphine consumption.

In present study, both RD and HD remifentanil reduced the mechanical pain threshold in the forearm, a site distant to the surgical site. Echevarria and colleagues recently reported that 0.2 μg kg^−1^min^−1^ remifentanil decreased the mechanical sensory threshold in the forearm after septorhinoplasty, suggesting increased pain sensitivity [11]. Schmidt and colleagues reported that in patients with no pain at the surgical site, intraoperative administration of HD remifentanil (0.4 μg kg^−1^min^−1^) increased sensitivity of painful pressure stimulation [12]. In agreement with these previous studies, intraoperative infusion of RD remifentanil in our study enhanced mechanical pain sensitivity. However, intraoperative application of HD remifentanil also decreased the mechanical pain threshold in the forearm at 24 h postoperatively. This finding is inconsistent with reduced morphine consumption and lower postoperative VAS scores with intraoperative HD remifentanil. Why HD remifentanil relieved postoperative pain in one situation and induced mechanical pain hypersensitivity in another situation is unclear.

Nevertheless, recent studies of spinal LTP induction by C-fiber-evoked field potential as a synaptic model of hyperalgesia to investigate the effect of HD remifentanil on hyperalgesia indicated that HD remifentanil may have opposing effects on spinal LTP induction in naïve or noxious-stimulated rats [6], [13]. In naïve rats, remifentanil withdrawal after 1 h of infusion induced spinal LTP in C-fiber evoked field potentials. The hyperalgesic effect of remifentanil is mainly mediated by activation of heterotrimeric guanine nucleotide-binding proteins, NMDA receptors, and increasing Ca^2+^ concentrations [2], [6], [13]. Because mechanical pain thresholds in the forearm mainly reflects a site without noxious stimuli, mimicking the experimental study in naïve rats [13], the findings in our study in which remifentanil (>0.2 μg kg^−1^min^−1^) induced mechanical hypersensitivity in the forearm are consistent with animal studies. NMDA receptor antagonists, ketamine, magnesium sulfate and propofol reduced remifentanil-induced hypersensitivity, further suggesting the involvement of the NMDA receptor in opioid-induced mechanical pain hypersensitivity [1], [3], [4], [14]. In contrast, in the presence of noxious stimuli, a similar experimental protocol inhibited the induction of spinal LTP by C-fiber stimulation probably by inhibiting the phosphorylation of α-amino-3-hydroxy-5-methyl-4-isoxazolepropionic acid (AMPA) receptors. AMPA receptors are well known to mediate spinal sensitization of incisional pain [15], [16]. In the present study, reduced morphine consumption and lower postoperative VAS scores in the HD group may be mediated by inhibiting the activation of spinal AMPA receptors. Taken together, differential effects of HD remifentanil on postoperative pain may be mediated by different mechanisms. In this regard, HD remifentanil may induce pain hypersensitivity in the intact site (such as the forearm) via activating NMDA receptor signaling while attenuating pain at the surgical site via inhibition of AMPA receptor signaling.

The persistent analgesic effect of HD remifentanil suggests that intraoperative infusion of HD opioids may be a novel strategy for postoperative pain control. This method may be simpler to undertake during surgery and postoperative treatment may not be required. In addition, reducing the use of other analgesics may also reduce side effects such as respiratory depression and itching. However, the development of hyperalgesia and the cost of remifentanil may limit potential clinical applications of remifentanil. Further studies are required to examine the effect of HD remifentanil on postoperative pain in other more painful surgeries such as coronary artery bypass graft. In the latter case, the potential attenuation of postoperative pain by HD remifentanil would greatly improve the quality of postoperative recovery and this has obvious clinical significance.

Of note, in the present study, we used propofol for maintenance of anesthesia. Numerous studies suggest that propofol, as a NMDA receptor antagonist, may attenuate the development of acute postoperative hyperalgesia by remifentanil withdrawal, and thereby reduce postoperative morphine consumption [17], [18]. However, there was no significant difference in propofol consumption between the two groups in the present study which may exclude possible bias of the anti-hyperalgesic effect of propofol. It remains to be determined whether inhaled anesthetics influence the effect of intraoperative HD remifentanil on postoperative pain and further investigation is warranted.

In conclusion, the present study showed that compared to RD remifentanil, HD remifentanil attenuated postoperative pain by reducing postoperative VAS score and morphine consumption. Thus, intraoperative modulation of high dose opioids may offer a convenient and effective technique for postoperative pain control.

## Supporting Information

Checklist S1 **Supporting CONSORT checklist.**
(DOC)

Protocol S1 **Trial Protocol in Chinese submitted to the hospital ethical committee.**
(DOCX)

Protocol S2 **English copy of trial protocol.**
(DOCX)
