# Supplementary material for: Effect of Intraoperative High-Dose Remifentanil on Postoperative Pain: A Prospective, Double Blind, Randomized Clinical Trial
Source: PLoS One. 2014 Mar 25;9(3):e91454. doi: 10.1371/journal.pone.0091454 (PMC3965388; doi:10.1371/journal.pone.0091454)
Supplement: Protocol S1 — Trial Protocol in Chinese submitted to the hospital ethical committee. (DOCX) [file pone.0091454.s002.docx]

大剂量瑞芬太尼对甲状腺切除术后患者疼痛的影响：随机、双盲、对照研究计划

**摘要**

研究背景：瑞芬太尼是一个广泛应用于临床镇痛的μ受体激动药。大量研究表明，0.2-0.4μg kg^-1^ min^-1^瑞芬太尼与小剂量（0.05μg kg^-1^min^-1^）比，可以导致术后痛觉超敏。而最近基础研究发现使用更大剂量的瑞芬太尼可以减少痛觉超敏的发生。我们的研究是通过比较持续泵入超大剂量瑞芬太尼和大剂量瑞芬太尼对甲状腺切除术患者VAS、机械痛敏、吗啡使用量等指标的影响，研究术中使用超大剂量瑞芬太尼对患者术后疼痛的影响，以及是否会抑制术后痛觉超敏。

研究方法：收集从2012年12月至2013年3月在中南大学湘雅二医院行甲状腺手术的患者。通过随机、对照，前瞻性研究，把60例甲状腺切除术患者分为大剂量组和超大剂量组。大剂量组和超大剂量组患者术中分别给予0.2μg kg-1min-1和1.2μg kg-1min-1瑞芬太尼，以及根据BIS值通过TCI调整丙泊酚用量，使BIS值维持在40-60。比较两组患者术后2h、18-24h机械痛阈变化，15min、30min、45min、60min、90min、120min、18-24h视觉疼痛模拟评分，以及苏醒时间、拔管时间，术后吗啡用量。

讨论：我们是第一个采用超大剂量瑞芬太尼的临床研究，我们的数据可以直接提示超大剂量瑞芬太尼是否可以抑制术后痛觉超敏。

注册号：

**ClinicalTrials.gov Identifier:** NCT01761149

**引言**

瑞芬太尼是一个超短效的μ受体激动药，由于半衰期短，苏醒快，无蓄积作用而被广泛应用于临床镇痛。痛觉超敏（hyperalgesia）是指痛阈的减退或者对相同痛觉刺激反应增强。大量实验表明，瑞芬太尼，和其他μ受体激动药一样，在停药后可以诱导继发性痛觉超敏，并且痛觉超敏与瑞芬太尼剂量呈一定正相关，即大剂量瑞芬太尼比小剂量瑞芬太尼更容易导致痛觉超敏 ^[^[^1^](#_ENREF_1)^,^ [^2^](#_ENREF_2)^]^。研究表明，大剂量瑞芬太尼（0.4μg Kg^-1^min^-1^）增加术后疼痛以及吗啡的用量，而小剂量瑞芬太尼（0.05μg Kg^-1^min^-1^）对术后痛阈及吗啡用量没有明显改变。另一项最近的研究表明，0.2μg Kg^-1^min^-1^瑞芬太尼比0.05μg Kg^-1^min^-1^可以使切口周围的痛觉更敏感，视觉模拟评分（visual analogue scale VAS）更高。此外，也有人研究发现术中给予0.3μg Kg^-1^min^-1^瑞芬太尼可以降低切口远端QST。研究表明，0.2-0.4μg Kg^-1^min^-1^瑞芬太尼与小剂量（0.05μg Kg^-1^min^-1^）比，可以增加术后疼痛^[^[^3^](#_ENREF_3)^]^及吗啡使用量^[^[^4^](#_ENREF_4)^]^，降低切口远端痛阈（Quantitative sensory threshold，QST）^[^[^5^](#_ENREF_5)^]^，导致术后痛觉超敏。

阿片类药物诱导的痛觉超敏（opioid-induced hyperalgesia OIH）的发生机制现在还不明了。脊髓背角浅层长时程强化（long-term potentiation , LTP）是OIH与损伤诱导的痛觉超敏共同机制^[^[^1^](#_ENREF_1)^,^ [^6-8^](#_ENREF_6)^]^。当停止使用阿片类药物时，脊髓背角浅层神经突触后膜活化^[^[^9^](#_ENREF_9)^]^，NMDA(N-methyl-D-aspartate)受体被激活^[^[^10-12^](#_ENREF_10)^]^，胞质内钙离子通过钙离子释放通道（RYR离子通道）释放增加，LTP形成^[^[^9^](#_ENREF_9)^]^。动物和临床实验均表明，使用NMDA受体拮抗剂如二氧化氮，硫酸镁，氯胺酮等可以阻止0.2-04μg Kg^-1^min^-1^瑞芬太尼造成的术后痛觉超敏^[^[^3-5^](#_ENREF_3)^,^ [^13^](#_ENREF_13)^]^，说明LTP及NMDA受体是OIH发生的重要机制。

Drdla等最近发现使用超大剂量瑞芬太尼可以逆转大鼠C-神经纤维LTP形成^[^[^14^](#_ENREF_14)^]^。在大鼠模型上，超大剂量（450μg Kg^-1^hour^-1^）瑞芬太尼可以抑制由低频刺激造成的坐骨神经C-神经纤维LTP形成，而小剂量（225μg Kg^-1^hour^-1^）瑞芬太尼却不能抑制C-神经纤维LTP形成。更进一步研究发现，持续泵入1小时超大剂量瑞芬太尼，可以阻止由辣椒素诱导的机械痛觉超敏长达6个小时。这项研究发现表明，超大剂量瑞芬太尼可能持续抑制由损伤造成的痛觉超敏，而不是造成更明显的痛觉超敏。同样，另一项研究发现，在大鼠神经病理性疼痛模型，持续泵入20μg Kg^-1^hour^-1^瑞芬太尼20分钟以上，可以抑制小鼠热痛觉超敏^[^[^11^](#_ENREF_11)^]^。提示超大剂量瑞芬太尼可以抑制OIH的发生。

我们的研究目的是通过比较持续泵入超大剂量瑞芬太尼和大剂量瑞芬太尼对甲状腺切除术患者VAS、QST、吗啡使用量等指标的影响，研究术中使用超大剂量瑞芬太尼对患者术后疼痛的影响，以及是否会抑制术后痛觉超敏。

**研究方法：**

选择2012年12月至2013年3月在我院行全麻下甲状腺全切术患者，年龄18-60岁，性别不限，ASA I-II级，BMI 18-30。随机分成超大剂量（UHD）组和大剂量（RHD）组。排除近期有慢性疼痛、药物滥用、酗酒、有心理疾病、长期或短期内（48小时内）使用镇痛药物患者。有颈部手术史及再次手术患者也排除。

采用TCI模式诱导，使丙泊酚及瑞芬太尼分别达到3-5 ug/ml和1ng/ml，当BIS值稳定在40-50，给予0.15mg/ml顺式阿曲库铵诱导插管。插管后，给与机械通气，维持氧浓度40%，不给予任何呼吸麻醉药。术中麻醉维持使用丙泊酚和瑞芬太尼，使BIS值维持在40-60。实验开始前，采用随机数字表法，患者被随机分到UHD和RHD组，每组30人。采用双盲法，在手术当天由一位不参与患者麻醉操作及术后评估的人，根据分组配置瑞芬太尼针。而患者和参与术后VAS评分、吗啡用量统计、QST等评估的，不知道分组结果。为了防止意外，在特殊情况下，实验设计者可以揭露分组结果并给予相应补救措施。麻醉维持阶段具体分组如下：UHD组：瑞芬太尼1.2μg kg^-1^min^-1^； RHD组：0.2μg kg^-1^min^-1^瑞芬太尼；从切皮开始，持续泵入瑞芬太尼至手术皮肤缝合完毕。当患者收缩压(systolic blood pressure SBP)低于80mmHg或平均动脉压（mean arterial pressure MAP）低于60mmHg时，给予5mg麻黄碱，当心率小于50次/分时，给予0.3mg阿托品。当患者VAS≥4时，给予吗啡镇痛。记录吗啡消耗量以及副作用。

**
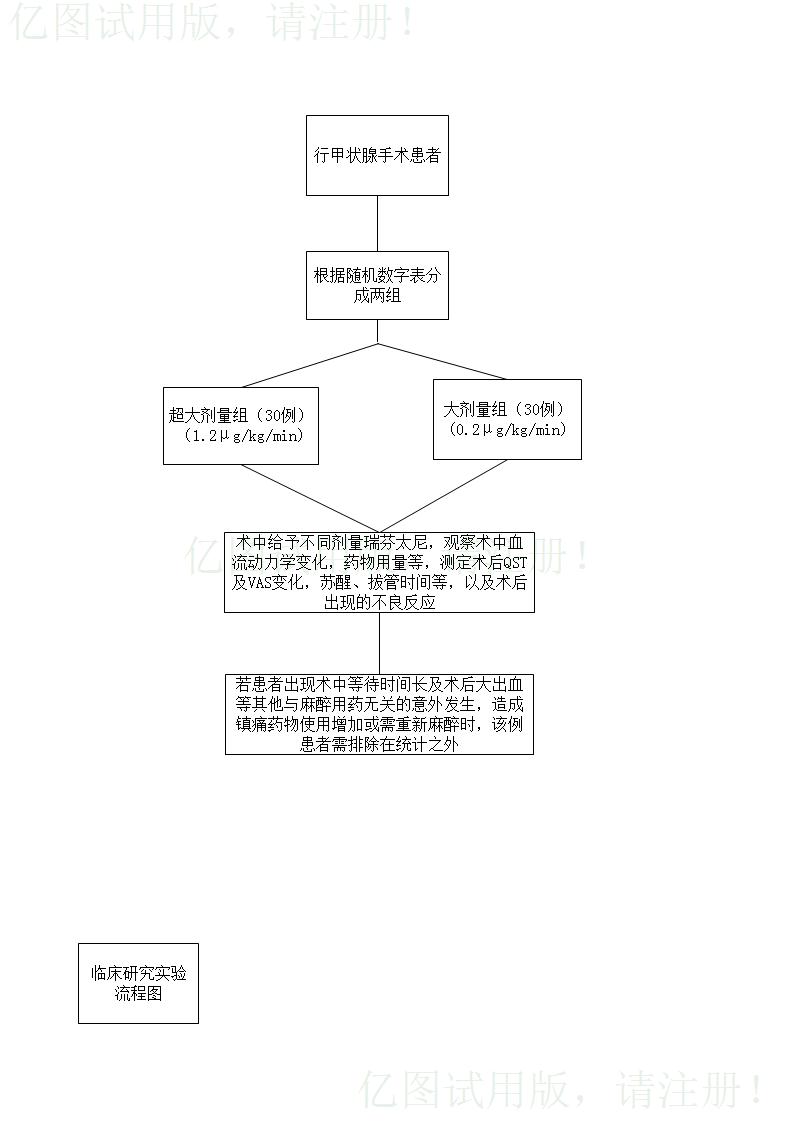
**

**观测指标：**

1、QST 通过VON-Frey探针测量前臂内侧大约3×3cm^2^区域痛阈，从小探针至大探针，轻轻用力，直至垂直于皮肤的探针弯曲，保持探针弯曲1-1.5秒，每次间隔15秒以上，以患者第一次感觉痛为所测的QST。术前1天测量基础值，术后2小时、18-24小时测量

2、VAS 通过视觉模拟评分表，0分为无痛，10分为最痛，测量患者15min, 30min,45min,60min,90min,120min,18-24h VAS值。

3、血流动力学 观察患者入手术室SBP、DBP、MAP、BIS，氧饱和度的基础值，以及插管前1min,插管后1min,5min,10min,20min,40min,手术结束、拔管后5min的值。

4、吗啡总用量 术后24小时内吗啡总用量（当VAS≥4分时，给予吗啡0.05mg/Kg）。

5、苏醒时间和拔管时间 停药后至苏醒时间及拔出气管插管时间

6、有无呕吐、震颤等副作用

**样本量估计**

通过我们的预试验中，超大剂量瑞芬太尼及大剂量瑞芬太尼组患者前臂术后24小时的QST分别是3.0±0.8和3.6±0.8（经过自然对数处理）。我们采用β误差为80%，α值为0.05作为两组有差别的检验水平，估计样本量为每组28例，我们每组纳入30例进行研究，以防有退出研究过程的病例。

**数据处理及统计方法**

所有数据采用SPSS13.0进行统计。一般资料采用平均数±方差表示，QST值经自然对数转换后用平均值加减方差表示。患者年龄、体重、身高、BMI、手术时间、术中丙泊酚使用总量采用ANOVA分析。患者性别、ASA分级、术中使用肾上腺素及麻黄素次数，是否发生术后并发症等采用卡方分析。术中血流动力学，BIS，VAS等值采用重复测量实验ANOVA进行对比分析。

**预期结果**

1.两组在性别、年龄、体重、BMI，ASA分级比较，无明显差异（p>0.05），具有可比性。

2.两组在术后2小时及术后24小时，QST比较有显著性差异(p<0.05)，超大剂量组QST评分低于大剂量组，机械痛阈下降不明显，痛觉过敏较大剂量组减轻。

3.两组在术后VAS评分，有显著性差异(p<0.05)，超大剂量组VAS评分较低。

4.两组在术中血流动力学上比较无明显差异(p>0.05)，说明超大剂量瑞芬太尼对全麻患者血流动力学影响不明显。

5.两组在术中肾上腺素、麻黄素比较无明显差异(p>0.05)。

6.两组比较，术后苏醒时间及拔管时间无明显差异(p>0.05)。

7.两组比较，术后24小时吗啡使用量有明显差异，超大剂量组吗啡用量少(p<0.05)。

**讨论**

我们是研究术中应用超大剂量瑞芬太尼对术后患者疼痛的影响。通过比较超大剂量组及大剂量组瑞芬太尼患者术后QST及VAS评分，可以观察术中超大剂量瑞芬太尼是否抑制术后痛觉超敏的发生，不仅是对动物实验的进一步验证和补充，还为临床镇痛提供一个有力的依据。

但是我们的研究也还存在一定的问题，伦理委员会不支持我们测量患者伤口附近的机械痛阈，给我们的研究带来了一些隐患。同时，患者的合作程度，VAS的主观性强等给我们的数据收集及统计带来一定的麻烦。

**分工**

徐军美、戴茹平：此研究主要负责人，参与研究的设计、资料的统计等工作。

陈艳萍、张燕玲：负责研究的具体实施方案设计，麻醉操作的执行者。

欧鹏： 负责研究对象的评估、术后疼痛评分等，以及后续部分统计工作等。

1. Angst, M.S. and J.D. Clark, *Opioid-induced hyperalgesia: a qualitative systematic review.* Anesthesiology, 2006. **104**(3): p. 570-87.

2. Burkle, H., S. Dunbar, and H. Van Aken, *Remifentanil: a novel, short-acting, mu-opioid.* Anesth Analg, 1996. **83**(3): p. 646-51.

3. Song, J.W., et al., *Magnesium sulfate prevents remifentanil-induced postoperative hyperalgesia in patients undergoing thyroidectomy.* Anesth Analg, 2011. **113**(2): p. 390-7.

4. Joly, V., et al., *Remifentanil-induced postoperative hyperalgesia and its prevention with small-dose ketamine.* Anesthesiology, 2005. **103**(1): p. 147-55.

5. Echevarria, G., et al., *Nitrous oxide (N(2)O) reduces postoperative opioid-induced hyperalgesia after remifentanil-propofol anaesthesia in humans.* Br J Anaesth, 2011. **107**(6): p. 959-65.

6. Mayer, D.J., et al., *Cellular mechanisms of neuropathic pain, morphine tolerance, and their interactions.* Proc Natl Acad Sci U S A, 1999. **96**(14): p. 7731-6.

7. Ikeda, H., et al., *Synaptic amplifier of inflammatory pain in the spinal dorsal horn.* Science, 2006. **312**(5780): p. 1659-62.

8. Sandkuhler, J., *Models and mechanisms of hyperalgesia and allodynia.* Physiol Rev, 2009. **89**(2): p. 707-58.

9. Drdla, R., et al., *Induction of synaptic long-term potentiation after opioid withdrawal.* Science, 2009. **325**(5937): p. 207-10.

10. Manering, N.A., et al., *High-dose remifentanil prevents development of thermal hyperalgesia in a neuropathic pain model.* Br J Anaesth, 2013. **110**(2): p. 287-92.

11. Zhao, M. and D.T. Joo, *Enhancement of spinal N-methyl-D-aspartate receptor function by remifentanil action at delta-opioid receptors as a mechanism for acute opioid-induced hyperalgesia or tolerance.* Anesthesiology, 2008. **109**(2): p. 308-17.

12. Chu, L.F., M.S. Angst, and D. Clark, *Opioid-induced hyperalgesia in humans: molecular mechanisms and clinical considerations.* Clin J Pain, 2008. **24**(6): p. 479-96.

13. Lee, L.H., M.G. Irwin, and S.K. Lui, *Intraoperative remifentanil infusion does not increase postoperative opioid consumption compared with 70% nitrous oxide.* Anesthesiology, 2005. **102**(2): p. 398-402.

14. Drdla-Schutting, R., et al., *Erasure of a spinal memory trace of pain by a brief, high-dose opioid administration.* Science, 2012. **335**(6065): p. 235-8.
