# Supplementary material for: Effect of Intraoperative High-Dose Remifentanil on Postoperative Pain: A Prospective, Double Blind, Randomized Clinical Trial
Source: PLoS One. 2014 Mar 25;9(3):e91454. doi: 10.1371/journal.pone.0091454 (PMC3965388; doi:10.1371/journal.pone.0091454)
Supplement: Protocol S2 — English copy of trial protocol. (DOCX) [file pone.0091454.s003.docx]

Effect of Higher Doses of Remifentanil on Postoperative Pain in Patients Undergoing Thyroidectomy：a prospective, double blind, randomized controlled trial

**Background:** Intraoperative administration of remifentanil is well known to exacerbate postoperative pain. However, experimental studies have demonstrated that higher dose remifentanil, in contrast to regular dose remifentanil, may attenuate hyperalgesia by erasing spinal sensitization. It remains elusive whether the phenomenon that large dose remifentanil exerts analgesia is also occurred in the surgical pain clinically.

**Purpose:** The present study hypothesizes that intraoperative large dose of remifentanil attenuates postoperative pain and aimed to test this hypothesis.

**Study Design:**

- **Allocation:** Randomized;
- **Intervention Model:** Parallel Assignment;
- **Masking:** Double blind (Investigator and outcome assessor are blind the assignment of the groups)

**Estimated Enrollment:** Sixty patients

**Group Assignment:**

- **Group I:** Regular group (RD): Patients received 0.2μg kg^-1^min^-1^ intraoperatively;
- **Group II:** High dose group (HD): Patients received 1.2 μg kg^-1^min^-1^ intraoperatively

|  |  |
| --- | --- |

**Criteria**

- **Inclusion Criteria:**
- ASA Grade I or II
- Age 18-60 years old
- BMI<30
- **Exclusion Criteria:**
- Not consent to participate into the trials
- Chronic pain,
- Used pain killer,
- Undergoing operation previously
- Diabetes or the other diseases affecting the pain sensation
- Difficult intubation;
- Unexpected surgical complication such as bleeding;
- Surgery interruption
- Psychiatric disorders;
- Drug or alcohol abuse

**Primary Outcome Measures:**

- **Visual analogue score (VAS) postoperatively**: VAS is widely used to assess postoperative pain. It will be divided as 10 points. Zero refers to no pain and ten refers to extremely pain. Based on this way, we can know the difference of postoperative pain in these two different groups.
- **Secondary Outcome Measures:**
- Cumulative Postoperative Opioid Consumption: When the patients described VAS ≥4, i.v. morphine will be infused at 0.05 mg/kg at 15 min intervals until the VAS <4.
- Mechanical Pain Threshold: A set of 10 hand-held Von Frey monofilaments calibrated to deliver an increasing force on the skin from 4 g to 300 g, is used for this purpose (Touch-test Sensory Evaluator, North Coast Medical Inc, CA, USA) as described previously. With the patients relaxed and eyes closed, in quiet surroundings, the filaments are placed perpendicularly against the skin surface until the hairs bent, and are held in place for 1–1.5 s. An interval of 15 s is allowed between trials. The mechanical pain threshold is defined as the smallest force that is felt painful by the patient.

**Anesthesia Procedure:**

Anesthesia is induced with continuous propofol infusion and remifentanil by target controlled infusion and 1 ng ml^-1^ respectively, based on the BIS value. Once the BIS value is below 60, 0.15 mg kg^-1^ cisatracurium is administered to facilitate tracheal intubation. After tracheal intubation, the patients are ventilated with 40% oxygen without any inhaled anesthetics. Maintenance of anesthesia is achieved with propofol only by target controlled infusion model to maintain the BIS value between 40 and 60.

In the case of hypotension, defined by a systolic arterial pressure less than 80 mmHg or a mean arterial pressure less than 60 mmHg, i.v. 5 mg ephedrine is given and additional intravenous fluids are also given as deemed appropriate by the responsible anesthesiologist. If heart rate is less than 50 per min, then 0.3 mg atropine is administered to maintain the heart rate above 50 per min.

**Statistical analysis**

Based on the result of preliminary study, the estimated sample size is 28 patients per group with a inβ-risk of 80% at an α-level of 0.05 for detecting a difference in mechanical pa threshold at least of 0.8 at 24 hours after operation when compared to baseline. Thus, we plan to enroll 30 patients per group to investigate the effect of HD remifentanil on mechanical pain sensation with allowing for possible dropouts.

Age, weight, height, BMI, duration of surgery and intraoperative propofol consumption will be analyzed by unpaired *t* test. Hemodynamic parameters including systolic (SBP), diastolic (DBP) and mean arterial pressure (MAP), heart rate (HR), BIS scale, mechanical pain threshold and the VAS score will be analyzed by two-way ANOVA for inter-group comparison. In cases of statistical significance, post hoc tests are conducted with Bonferroni adjustment. The χ^2^ test is used to compare gender, ASA level, intraoperative ephedrine or morphine consumption, and postoperative complications (postoperative nausea and vomiting, respiratory depression, shivering). All analyses will be performed using SPSS 13.0 and GraphPad Prism 5.
